# Supplementary material for: LncMyoD Promotes Skeletal Myogenesis and Regulates Skeletal Muscle Fiber-Type Composition by Sponging miR-370-3p
Source: Genes (Basel). 2021 Apr 17;12(4):589. doi: 10.3390/genes12040589 (PMC8072939; doi:10.3390/genes12040589)
Supplement: Supplementary file 1 [file genes-12-00589-s001.zip › supplement filesτÜäσë»μ£1⁄4/Supplement Table1.docx]

| **Gene** | **Primer sequence (5`→ 3`)** |
| --- | --- |
| *MyoD* | F: AGACTTCTATGATGACCCGTGTT |
|  | R: TCAGCGTTGGTGGTCTTGC |
| *MyoG* | F: GCCCAGTGAATGCAACTCCCACA |
|  | R: CAGCCGCGAGCAAATGATCTCCT |
| *myf5* | F: GAGCTGCTGAGGGAACAGGTGGAGA |
|  | R: GTTCTTTCGGGACCAGACAGGGCTG |
| *MyHC* | F: CGCAAGAATGTTCTCAGGCT |
|  | R: GCCAGGTTGACATTGGATTG |
| *Cox2* | F:GCCGACTAAATCAAGCAACA |
|  | R:CAATGGGCATAAAGCTATGG |
| *Mterf1* | F: AGAGGCGGAAGTGAAAGGTG |
|  | R:AAGTTGCTCAGCAGGTCCTC |
| *Tfam* | F:CCAGGAGGCAAAGGATGATTCG |
|  | R:CCAACTTCAGCCATCTGCTCTTCCC |
| *Cox5b* | F:CTGGACCCATACAATATGCTACCTCC |
|  | R:ATCGCTGACTCTCGCCTTTGTG |
| *Cox8b* | F:GAACCATGAAGCCAACGACT |
|  | R:GCGAAGTTCACAGTGGTTCC |
| *MyHC IIx* | F:GGACCCACGGTCGAAGTTG |
|  | R:CCCGAAAACGGCCATCT |
| *MyHC IIb* | F:CAATCAGGAACCTTCGGAACAC |
|  | R:GTCCTGGCCTCTGAGAGCAT |
| *MyHC I* | F: ATGAGCTGGAGGCTGAGCA |
|  | R: TGCAGCCGCAGTAGGTTCTT |
| *MyHC IIa* | F: ATTCTCAGGCTTCAGGATTTGGTG |
|  | R: CTTGCGGAACTTGGATAGATTTGTG |
| *Tnni2* | F: GATGAGGAGAAGCGCAACAG |
|  | R: TTTCTCCTCTTCAGCCACGT |
| *Tnni1* | F: ATGCCGGAAGTTGAGAGGAA |
|  | R: CTGAAGGGCACTGAGAGACA |
| *Tnnc 2* | F: CGGCTCCATCGACTTTGAAG |
|  | R: AGCAGCTCATCGATCTCCTC |
| *Tnnc 1* | F: GCAAGGTGATGAGGATGCTG |
|  | R: GACTTCCCTTTGCTGTCGTC |
| *MRF4* | F: ATTCTTGAGGGTGCGGATTTCCTG |
|  | R: AAGACTGCTGGAGGCTGAGGCATC |
|  | R: TAGGACCATTACTGCCAGAGGA |
| *Lnc-MyoD* | F: GATGTGAATCCCGGTTCTGC |
|  | R: GGAGTAAGATGGGTGTGGCT |
| *P21* | F:GATGGCTTCGACACCATTCC |
|  | R:AGACGACACAGGTGAGGAAG |
| *CDK4* | F: GTCAGTTTCTAAGCGGCCTG |
|  | R: CACGGGTGTTGCGTATGTAG |
| *CDK6* | F: TCTGGCCAGCTCTTCATTCA |
|  | R: CGGCACACAGTAAGCTCTTC |
| *Cyclin D1* | F: GTTGCTGGAATTTTCGGGGT |
|  | R: AGCGTCCCTGTCTTCTTTCA |
| *Beta-actin* | F: CAGCCTTCCTTCTTGGGTAT |
|  | R: TGGCATAGAGGTCTTTACGG |
| *U6* | F: CTCGCTTCGGCAGCACA |
|  | R: AACGCTTCACGAATTTGCGT |
| *miR-370-3p* | GCCTGCTGGGGTGGAACCTGGT |
| *ACADSB* | F:CCCAACCTGCTTGTCTCCTTG |
|  | R: ATCCCTGGATCACCGATTTCT |

**Supplementary Table S1.** The primer sequences used for qRT-PCR. F: forward, R: reverse. U6 and *β-actin* were used as endogenous control genes for miRNA and mRNA, respectively.
