# Supplementary material for: LncMyoD Promotes Skeletal Myogenesis and Regulates Skeletal Muscle Fiber-Type Composition by Sponging miR-370-3p
Source: Genes (Basel). 2021 Apr 17;12(4):589. doi: 10.3390/genes12040589 (PMC8072939; doi:10.3390/genes12040589)

**Supplement Table2**

All plasmids are constructed on the psiCHECK vector. The following is the complete sequence and restriction diagram of each plasmid.

1. ACADSB-miR-370-3p MUT

| **Certificate of Analysis** | | | | | | | | |
| --- | --- | --- | --- | --- | --- | --- | --- | --- |
| **Gene Name** | acadsb-miR-370-3p MUT | | | | **Order No.** | Y0022475-1 | | |
| **Lot No.** | K5130/Y0022475-1 | | | | **Cloning Vector** | pSiCheck2 | | |
| **Cloning Sites** | XhoI-NotI | | | | **Insert Size** | 288 | | |
| **QC Results** | | | | | | | | |
| **Test Items** | | **Specifications** | | | | | | **Results** |
| **Insert Sequence** | | Insert sequence results consistent with target | | | | | | Pass |
| **Vector Sequence** | | Flanking sequence consistent with expected | | | | | | N/A |
| **ORF Across Junction** | | Correct and consistent with target | | | | | | N/A |
| **Restriction Digest** | | Expected fragment sizes observed | | | | | | Pass |
| **PCR Amplification** | | Correct without non - specific bands | | | | | | N/A |
| **DNA Quantity/Quality** | | Actual yield (by A 260 ) | | | | | | 4ug |
|  |  | Concentration (n/a if lyophilized) | | | | | | N/A |
|  |  | Purity (A 260/A280 = 1.8 - 2.0) | | | | | | Pass |
|  |  | # of Tubes | | | | | | 1 |
|  |  | Matrix | | | | | | ddH2O |
| **Endotoxin Test** | | Verified, <0.1 EU/µg (Endo-Free Preps Only) | | | | | | N/A |
| **Appearance** | | Clear, no visible particles | | | | | | Pass |
| **Label** | | Correct and white | | | | | | Pass |
| **Comments** | | NA | | | | | | |
| **Restriction Digestion Map** | | | | | | | | |
| 1 2 M | | | | | | | | |
| **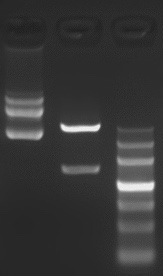** | | | **Lane1**: plasmid DNA  **Lane2**: Digested with  KpnI/NotI  **Lane M**: DNA Marker | | | | **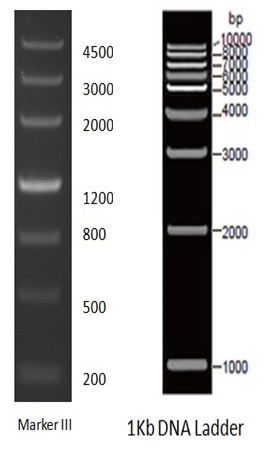** | |
| **Certified by: LIUTAO** | | | | **Date:**2019-11-12 | | | | |

**Insert sequence**

CTCGAGTGCAGAAGTCTGAATGCCAGGGCAGCAGCCCGCTGCTCCATTCTAGGAAAGCATTGCACATTCTAGGCAAAGGGCAGAACATCCCGCTGGCATTTCAAATAGCTCTTGAAAGGCAGCCCTCATCCATTCTTGTTCATGTCACAACAAGTTATAATAAAGCAGGCAGTGCCCTTGTGTTCGGGGTTAATTGGTTCTTTGGTTTGAGGCTTTTATGTCGTTTCTCTCAAGATCCACCTCTTACTCTTCAGCCGCTGTCCTATTCGCTTGACTTCTAGCGGCCGC

**Plasmids map**


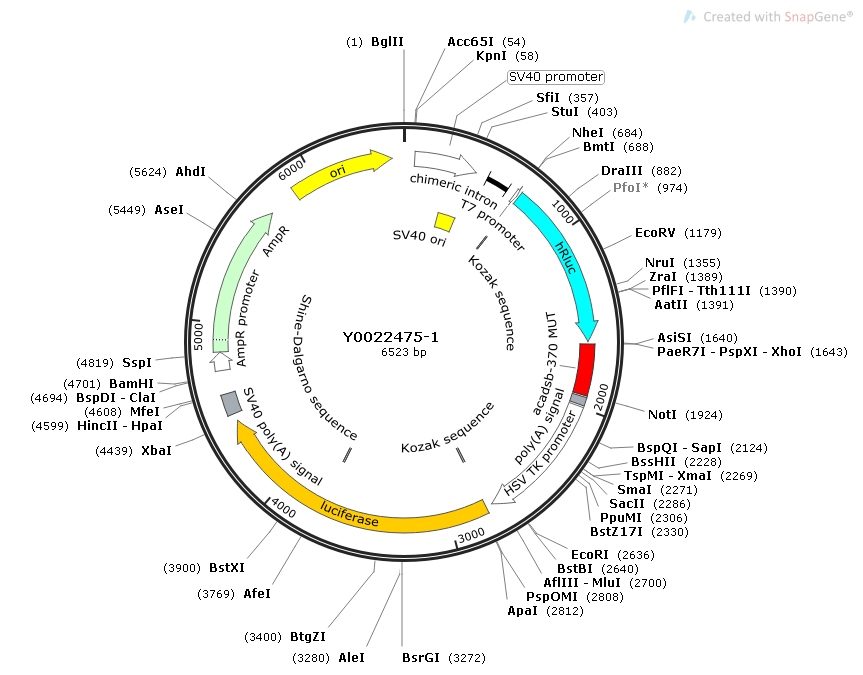


| **Certificate of Analysis** | | | | | | | | |
| --- | --- | --- | --- | --- | --- | --- | --- | --- |
| **Gene Name** | acadsb-miR-370-3p WT | | | | **Order No.** | Y0022475-2 | | |
| **Lot No.** | K5137/Y0022475-2 | | | | **Cloning Vector** | pSiCheck2 | | |
| **Cloning Sites** | XhoI-NotI | | | | **Insert Size** | 288 | | |
| **QC Results** | | | | | | | | |
| **Test Items** | | **Specifications** | | | | | | **Results** |
| **Insert Sequence** | | Insert sequence results consistent with target | | | | | | Pass |
| **Vector Sequence** | | Flanking sequence consistent with expected | | | | | | N/A |
| **ORF Across Junction** | | Correct and consistent with target | | | | | | N/A |
| **Restriction Digest** | | Expected fragment sizes observed | | | | | | Pass |
| **PCR Amplification** | | Correct without non - specific bands | | | | | | N/A |
| **DNA Quantity/Quality** | | Actual yield (by A 260 ) | | | | | | 4ug |
|  |  | Concentration (n/a if lyophilized) | | | | | | N/A |
|  |  | Purity (A 260/A280 = 1.8 - 2.0) | | | | | | Pass |
|  |  | # of Tubes | | | | | | 1 |
|  |  | Matrix | | | | | | ddH2O |
| **Endotoxin Test** | | Verified, <0.1 EU/µg (Endo-Free Preps Only) | | | | | | N/A |
| **Appearance** | | Clear, no visible particles | | | | | | Pass |
| **Label** | | Correct and white | | | | | | Pass |
| **Comments** | | NA | | | | | | |
| **Restriction Digestion Map** | | | | | | | | |
| / | | | | | | | | |
| **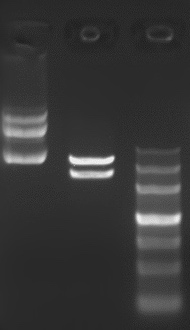** | | | **Lane1**: plasmid DNA  **Lane2**: Digested with  XhoI/XbaI  **Lane M**: DNA Marker | | | | **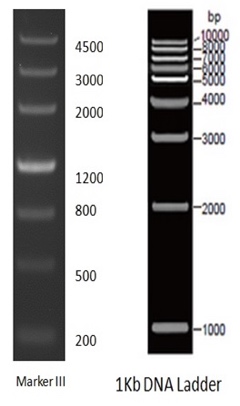** | |
| **Certified by: LIUTAO** | | | | **Date:**2019-11-12 | | | | |

2. ACADSB-miR-370-3p WT

**Insert sequence**

CTCGAGTGCAGAAGTCTGAATGCCAGGGCAGCAGCCCGCTGCTCCATTCTAGGAAAGCATTGCACATTCTAGGCAAAGGGCAGAACATCCCGCTGGCATTTCAAATAGCTCTTGAAAGGCAGCCCTCATCCATTCTTGTTCATGTCCAGCAGGGTTCAGCAGGAGCAGGCAGTGCCCTTGTGTTCGGGGTTAATTGGTTCTTTGGTTTGAGGCTTTTATGTCGTTTCTCTCAAGATCCACCTCTTACTCTTCAGCCGCTGTCCTATTCGCTTGACTTCTAGCGGCCGC

**Plasmids map**


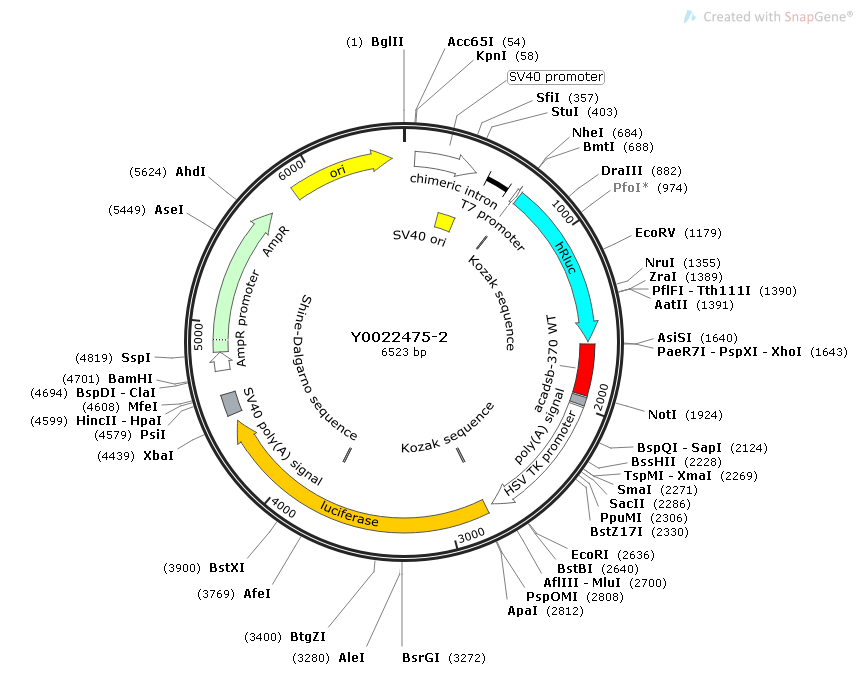


**3.lncMyoD-miR-370-3p MUT**

| **Certificate of Analysis** | | | | | | | | |
| --- | --- | --- | --- | --- | --- | --- | --- | --- |
| **Gene Name** | lnc-370 MUT | | | | **Order No.** | Y0022475-3 | | |
| **Lot No.** | K5941/Y0022475-3 | | | | **Cloning Vector** | pSiCheck2 | | |
| **Cloning Sites** | XhoI-NotI | | | | **Insert Size** | 296 | | |
| **QC Results** | | | | | | | | |
| **Test Items** | | **Specifications** | | | | | | **Results** |
| **Insert Sequence** | | Insert sequence results consistent with target | | | | | | Pass |
| **Vector Sequence** | | Flanking sequence consistent with expected | | | | | | N/A |
| **ORF Across Junction** | | Correct and consistent with target | | | | | | N/A |
| **Restriction Digest** | | Expected fragment sizes observed | | | | | | Pass |
| **PCR Amplification** | | Correct without non - specific bands | | | | | | N/A |
| **DNA Quantity/Quality** | | Actual yield (by A 260 ) | | | | | | 4ug |
|  |  | Concentration (n/a if lyophilized) | | | | | | N/A |
|  |  | Purity (A 260/A280 = 1.8 - 2.0) | | | | | | Pass |
|  |  | # of Tubes | | | | | | 1 |
|  |  | Matrix | | | | | | ddH2O |
| **Endotoxin Test** | | Verified, <0.1 EU/µg (Endo-Free Preps Only) | | | | | | N/A |
| **Appearance** | | Clear, no visible particles | | | | | | Pass |
| **Label** | | Correct and white | | | | | | Pass |
| **Comments** | | NA | | | | | | |
| **Restriction Digestion Map** | | | | | | | | |
| / | | | | | | | | |
| **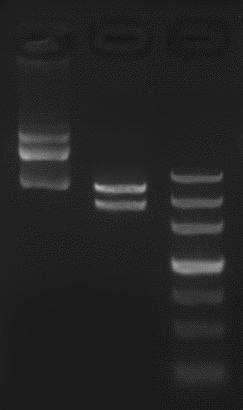** | | | **Lane1**: plasmid DNA  **Lane2**: Digested with  XbaI/XhoI  **Lane M**: DNA Marker | | | | **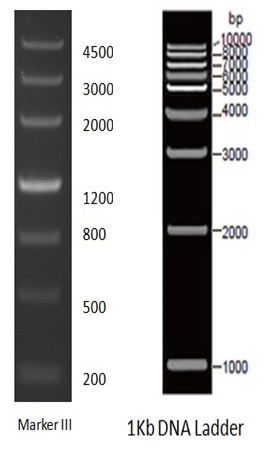** | |
| **Certified by: LIUTAO** | | | | **Date:**2019-11-16 | | | | |

**Insert sequence**

CTCGAGTCTGTCTGTGATGTGAACCAGATGATAGAGTTGTCACCCAAGGCAAGAAAGTAGCACCGGAGCCAGCATCAGAGGATACAAGCCTTGAAAGATGGGATGTGAATCCCGGTTCTGCCGCTGACTCGTGAGTGGCTTCAGACAGTAAAGTTTCAGGAGCAGACAACAAGCTCTGAAGGACACAAGGTGGCTTCCAGAGCACAGATGAAGATGTTGGCTGGGTTGGGGCTCATCTCAAGGCCTGACTGGGGAGAAGCCACACCCATCTTACTCCATCTTAACCTCGCGGCCGC

**Plasmids map**


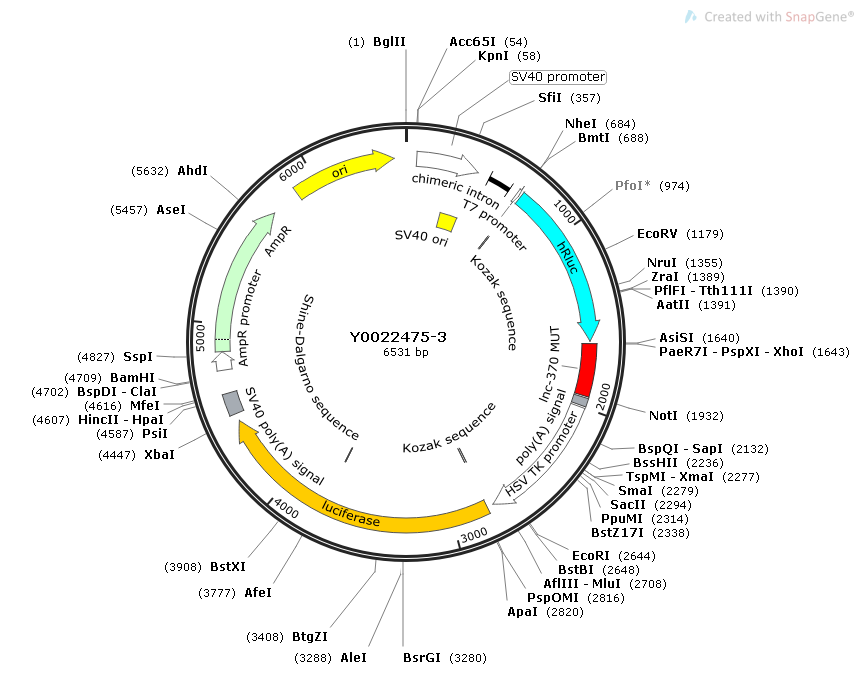


| **Certificate of Analysis** | | | | | | | | |
| --- | --- | --- | --- | --- | --- | --- | --- | --- |
| **Gene Name** | lnc-370 WT | | | | **Order No.** | Y0022475-4 | | |
| **Lot No.** | K5285/Y0022475-4 | | | | **Cloning Vector** | pSiCheck2 | | |
| **Cloning Sites** | XhoI-NotI | | | | **Insert Size** | 296 | | |
| **QC Results** | | | | | | | | |
| **Test Items** | | **Specifications** | | | | | | **Results** |
| **Insert Sequence** | | Insert sequence results consistent with target | | | | | | Pass |
| **Vector Sequence** | | Flanking sequence consistent with expected | | | | | | N/A |
| **ORF Across Junction** | | Correct and consistent with target | | | | | | N/A |
| **Restriction Digest** | | Expected fragment sizes observed | | | | | | Pass |
| **PCR Amplification** | | Correct without non - specific bands | | | | | | N/A |
| **DNA Quantity/Quality** | | Actual yield (by A 260 ) | | | | | | 4ug |
|  |  | Concentration (n/a if lyophilized) | | | | | | N/A |
|  |  | Purity (A 260/A280 = 1.8 - 2.0) | | | | | | Pass |
|  |  | # of Tubes | | | | | | 1 |
|  |  | Matrix | | | | | | ddH2O |
| **Endotoxin Test** | | Verified, <0.1 EU/µg (Endo-Free Preps Only) | | | | | | N/A |
| **Appearance** | | Clear, no visible particles | | | | | | Pass |
| **Label** | | Correct and white | | | | | | Pass |
| **Comments** | | NA | | | | | | |
| **Restriction Digestion Map** | | | | | | | | |
| / | | | | | | | | |
| **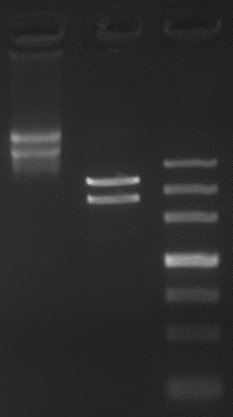** | | | **Lane1**: plasmid DNA  **Lane2**: Digested with  XbaI/XhoI  **Lane M**: DNA Marker | | | | **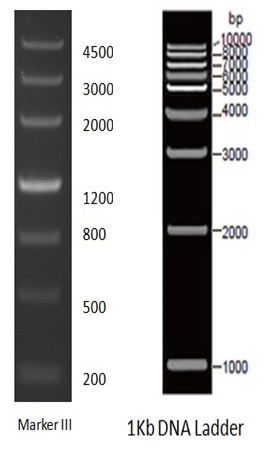** | |
| **Certified by: LIUTAO** | | | | **Date:**2019-11-13 | | | | |

**4. lncMyoD-miR-370-3p WT**

**Insert sequence**

CTCGAGTCTGTCTGTGATGTGAACCAGATGATAGAGTTGTCACCCAAGGCAAGAAAGTAGCACCGGAGCCAGCATCAGAGGATACAAGCCTTGAAAGATGGGATGTGAATCCCGGTTCTGCCGCTGACTCGTGAGTGGCTTCAGACAGTAAAGTTTCAGGAGCAGCAGCAGGGCTCTGAAGGACACAAGGTGGCTTCCAGAGCACAGATGAAGATGTTGGCTGGGTTGGGGCTCATCTCAAGGCCTGACTGGGGAGAAGCCACACCCATCTTACTCCATCTTAACCTCGCGGCCGC

**Plasmids map**


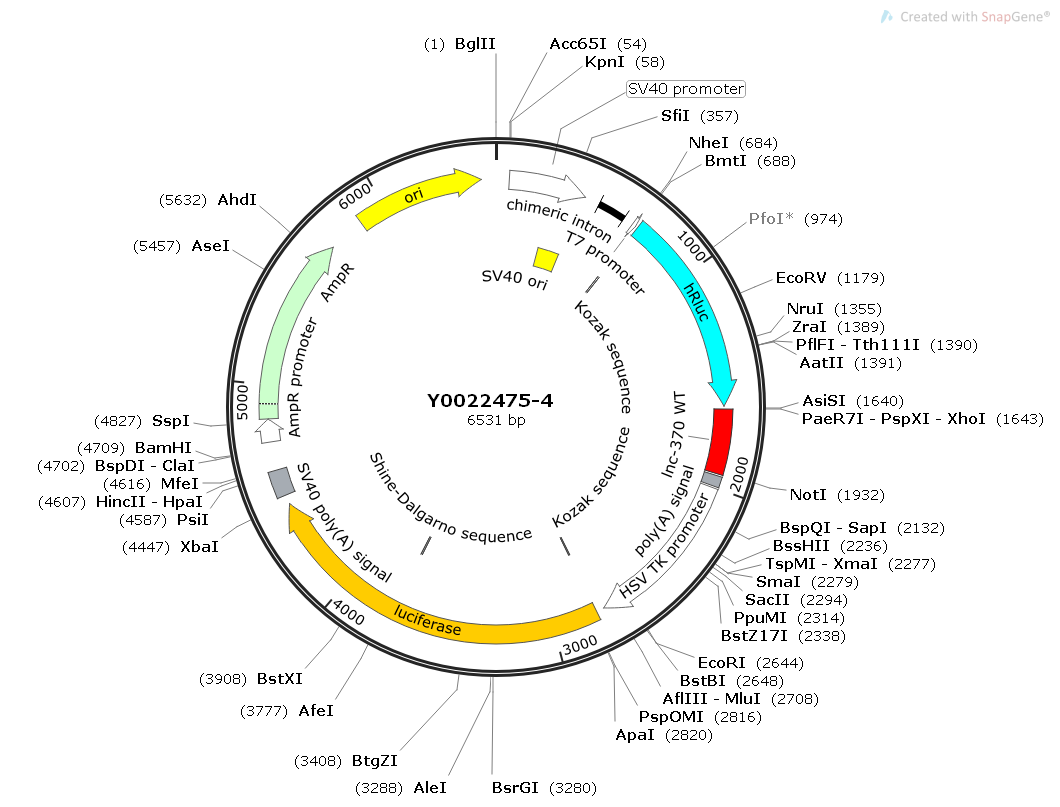

Supplement: Supplementary file 1 [file genes-12-00589-s001.zip › supplement filesτÜäσë»μ£1⁄4/Supplement Table2.docx]
