# Supplementary figures and images for: LncMyoD Promotes Skeletal Myogenesis and Regulates Skeletal Muscle Fiber-Type Composition by Sponging miR-370-3p
Source: Genes (Basel). 2021 Apr 17;12(4):589. doi: 10.3390/genes12040589 (PMC8072939; doi:10.3390/genes12040589)

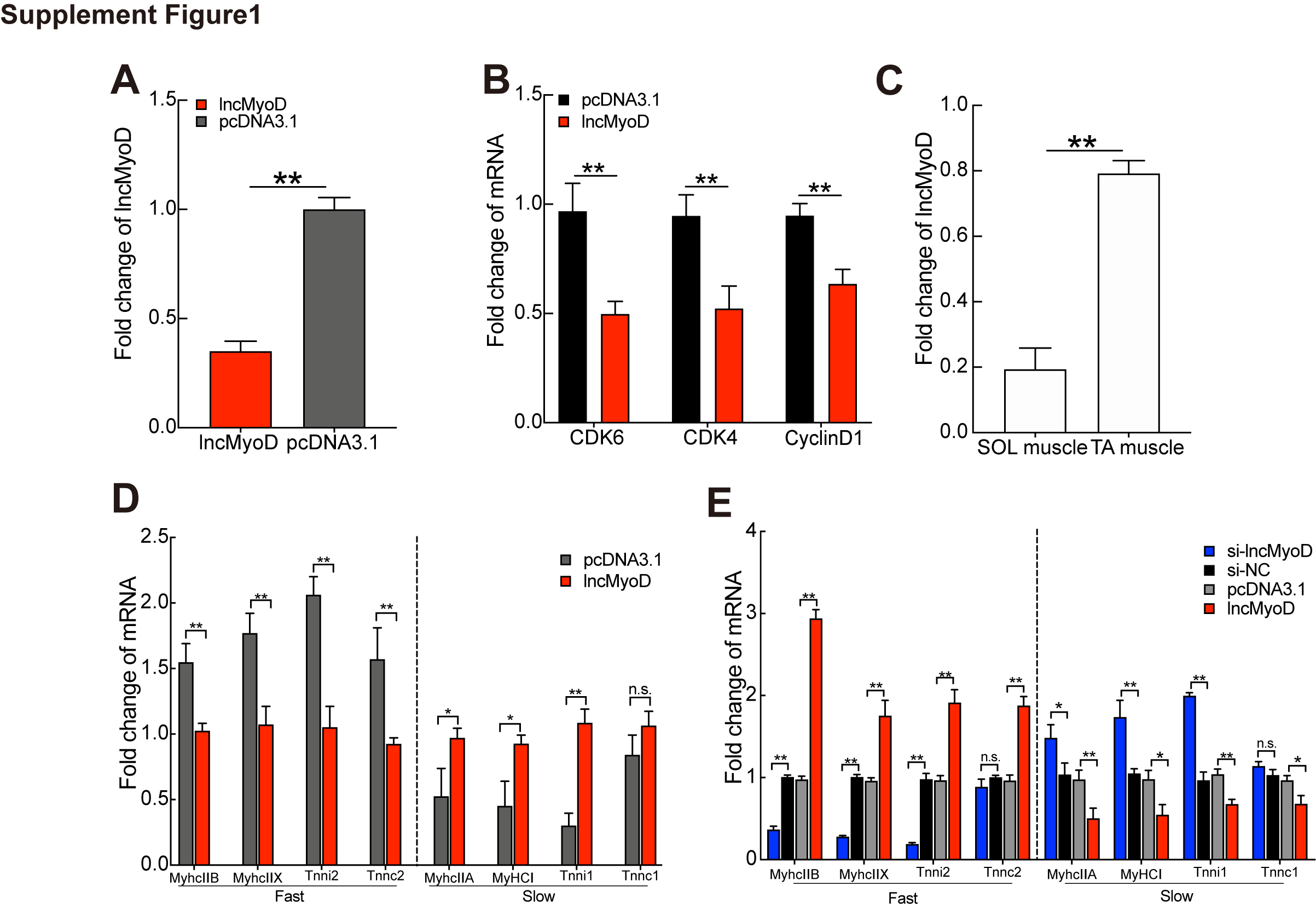

Supplement: Supplementary file 1 [file genes-12-00589-s001.zip › supplement filesτÜäσë»μ£1⁄4/Supplement Figure1.tif]

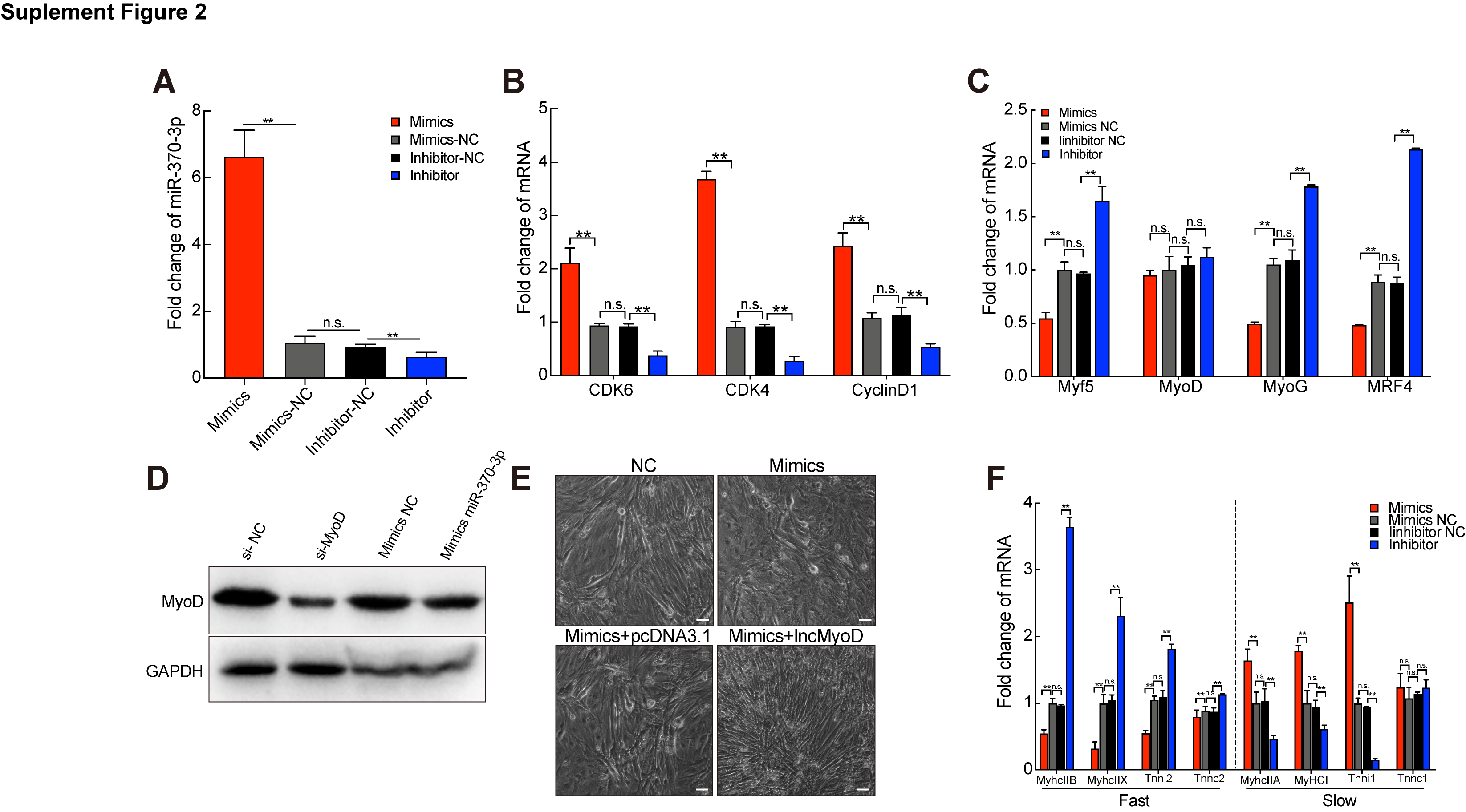

Supplement: Supplementary file 1 [file genes-12-00589-s001.zip › supplement filesτÜäσë»μ£1⁄4/Supplement Figure2.tif]

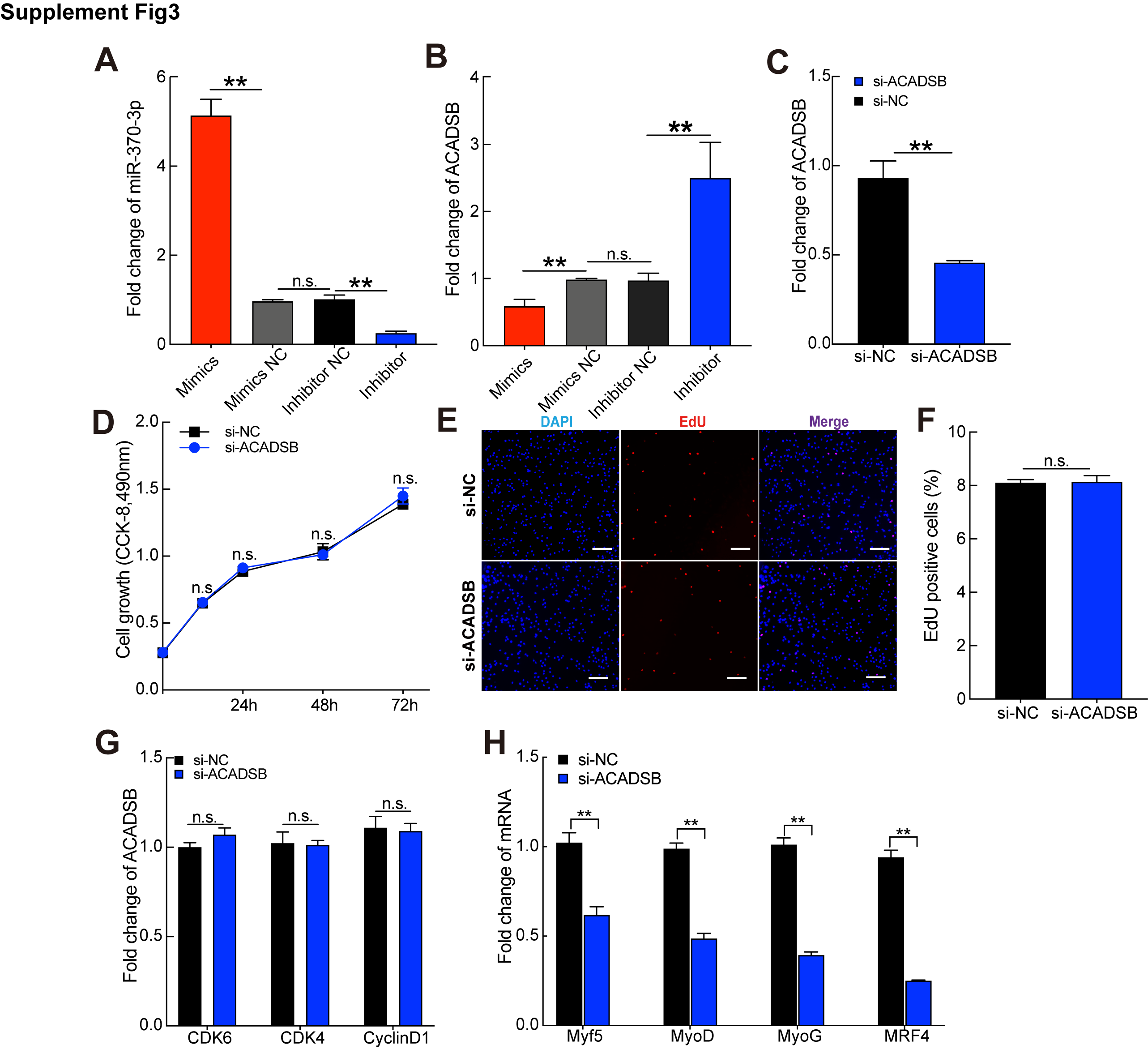

Supplement: Supplementary file 1 [file genes-12-00589-s001.zip › supplement filesτÜäσë»μ£1⁄4/Supplement Figure3.tif]
